# Association of Carbapenemase Gene Profiles with Ceftazidime–avibactam Resistance in Carbapenem-Resistant *Klebsiella pneumoniae*

**DOI:** 10.3390/ijms27209386

**Published:** 2026-10-21

**Authors:** Elif Tuğçe Güner, Ayfer Bakır, Cemal Çiçek, Murat Aral

**Affiliations:** Department of Medical Microbiology, University of Health Science Ankara Etlik City Hospital, Ankara 06170, Türkiye; dr.ayfer.bakir@gmail.com (A.B.); drcemal06@gmail.com (C.Ç.); aralmurat@hotmail.com (M.A.)

**Keywords:** *Klebsiella pneumoniae*, ceftazidime-avibactam, carbapenem resistance, carbapenemase, NDM

## Abstract

Carbapenem-resistant *Klebsiella pneumoniae* (CRKP) is a major healthcare threat, and carbapenemase genotype may influence ceftazidime–avibactam (CZA) susceptibility. This study evaluated the association between carbapenemase gene profiles and CZA resistance in CRKP isolates. In this retrospective laboratory-based study, 184 CRKP isolates (92 CZA-susceptible and 92 CZA-resistant) were analyzed. Carbapenemase genes were detected by multiplex real-time PCR, and gene distributions and profiles were compared according to CZA susceptibility. NDM was detected in 82.6% of CZA-resistant isolates but in none of the susceptible isolates (*q* < 0.001). All CZA-susceptible isolates carried a single carbapenemase gene, whereas 68.5% of resistant isolates carried two or more genes. NDM + OXA-48-like was the predominant profile among resistant isolates (63.0%), while OXA-48-like alone predominated among susceptible isolates (92.4%) (both *q* < 0.001). NDM showed 82.6% sensitivity and 100% specificity for discriminating CZA resistance, with an AUC of 0.913. Amikacin resistance was also higher among CZA-resistant isolates (60.9% vs. 31.5%; *q* < 0.001). NDM, particularly when co-occurring with OXA-48-like, was strongly associated with CZA resistance. Rapid carbapenemase genotyping may complement phenotypic susceptibility testing and support antimicrobial treatment decisions.

## 1. Introduction

*Klebsiella pneumoniae* is an important opportunistic pathogen responsible for various hospital-acquired infections, including bacteremia, pneumonia, urinary tract infections, and surgical site infections [1,2]. In recent years, the spread of carbapenem-resistant *K. pneumoniae* (CRKP) strains has become a major global health concern because of limited treatment options and its association with increased mortality [3,4]. Carbapenem resistance has been reported at substantial rates among hospital-acquired *K. pneumoniae* infections, with mortality in CRKP infections reaching approximately 42% [2,3].

Although carbapenemase production is the primary mechanism of carbapenem resistance in *K. pneumoniae*, porin loss, reduced outer membrane permeability, and efflux systems may also contribute to the resistance phenotype [5,6]. The most clinically relevant carbapenemases include KPC, NDM, VIM, IMP, and OXA-48-like enzymes. Among these, KPC represents serine β-lactamases; NDM, VIM, and IMP are metallo-β-lactamases (MBLs); and OXA-48-like enzymes belong to class D carbapenemases [4]. Türkiye is among the regions where OXA-48-like carbapenemases are widespread; however, other genotypes, including NDM and KPC, as well as the co-occurrence of multiple carbapenemases, have also been reported [7,8].

Ceftazidime–avibactam (CZA) is an important β-lactam/β-lactamase inhibitor combination used for the treatment of CRKP infections. Owing to the inhibitory activity of avibactam against serine β-lactamases, CZA exhibits high in vitro activity, particularly against isolates producing KPC and OXA-48-like enzymes [9,10,11]. In contrast, the inability of avibactam to inhibit MBLs such as NDM, VIM, and IMP substantially limits CZA activity against MBL-producing isolates [12,13]. Therefore, the carbapenemase genotype is an important molecular determinant for predicting CZA susceptibility.

However, CZA resistance cannot be explained solely by the presence of MBLs. Other mechanisms, including KPC variants and porin alterations affecting outer membrane permeability, may also contribute to the development of CZA resistance [11,14,15]. In particular, alterations in outer membrane proteins such as OmpK35/OmpK36 and LamB have been associated with the resistance phenotype [6,15]. Similarly, different molecular resistance mechanisms have been reported to emerge following CZA exposure in OXA-48-like-producing isolates [16,17]. These findings indicate that CZA resistance may arise through heterogeneous molecular mechanisms and highlight the importance of evaluating carbapenemase genotypes together with phenotypic susceptibility results.

Particularly in regions where OXA-48-like carbapenemases are widespread, the co-carriage of NDM and the presence of multiple carbapenemase profiles make it clinically and epidemiologically important to elucidate the relationship between CZA susceptibility and carbapenemase genotypes. This study aimed to investigate the association between CZA susceptibility status and carbapenemase genotypes and gene profiles among carbapenem-resistant *K. pneumoniae* isolates.

## 2. Results

### 2.1. Distribution of Carbapenemase Genes According to CZA Susceptibility Status

Of the 184 isolates, 92 (50.0%) were susceptible to CZA and 92 (50.0%) were resistant. At least one of the investigated carbapenemase genes was detected in all isolates in both groups.

Among CZA-susceptible isolates, OXA-48-like was the most frequently detected carbapenemase gene, being positive in 85/92 (92.4%) isolates. KPC was detected in 7/92 (7.6%) isolates, whereas NDM, OXA-23/58, OXA-51, IMP, and VIM were not detected. Among CZA-resistant isolates, NDM and OXA-48-like genes were detected in 76/92 (82.6%) and 74/92 (80.4%) isolates, respectively. KPC was detected in 6/92 (6.5%) isolates and OXA-23/58 in 1/92 (1.1%), whereas OXA-51, IMP, and VIM were not detected.

NDM positivity was significantly more frequent among CZA-resistant isolates than among CZA-susceptible isolates (82.6% vs. 0%; *p* < 0.001). In contrast, OXA-48-like positivity was more frequent among CZA-susceptible isolates (92.4% vs. 80.4%; *p* = 0.030). No significant difference in KPC positivity was observed between the two groups (7.6% vs. 6.5%; *p* = 1.000). After false discovery rate (FDR) correction, the difference in NDM positivity between CZA-resistant and CZA-susceptible isolates remained statistically significant (*q* < 0.001), whereas the difference observed for OXA-48-like was no longer statistically significant (*q* = 0.060) (Figure 1).

### 2.2. Association of Carbapenemase Gene Number and Gene Profiles with CZA Resistance

A single carbapenemase gene was detected in all CZA-susceptible isolates, and none of the susceptible isolates carried multiple carbapenemase genes. In contrast, a single carbapenemase gene was detected in 29/92 (31.5%) CZA-resistant isolates, whereas two or more carbapenemase genes were detected in 63/92 (68.5%). Among the resistant isolates, 61 (66.3%) carried two carbapenemase genes, while 2 (2.2%) carried three carbapenemase genes.

When carbapenemase gene profiles were evaluated, the OXA-48-like-only profile was the most frequently detected among CZA-susceptible isolates [85/92 (92.4%)], whereas the NDM + OXA-48-like combination was the most common profile among CZA-resistant isolates [58/92 (63.0%)]. The NDM-only profile was detected in 13/92 (14.1%) CZA-resistant isolates but was not observed in any CZA-susceptible isolate. The NDM + KPC combination was detected in three (3.3%) resistant isolates, while the NDM + OXA-48-like + KPC and NDM + OXA-48-like + OXA-23/58 combinations were each detected in one (1.1%) resistant isolate. After FDR correction, the OXA-48-like-only, NDM-only, and NDM + OXA-48-like profiles remained statistically significant (all *q* < 0.001), whereas no significant associations were observed for the other gene profiles (Table 1).

### 2.3. Performance of Carbapenemase Genes in Predicting CZA Resistance

When the performance of NDM presence in predicting CZA resistance was evaluated, the sensitivity was 82.6% (95% CI: 73.3–89.7), specificity was 100.0% (95% CI: 96.1–100.0), positive predictive value (PPV) was 100.0% (95% CI: 95.3–100.0), and negative predictive value (NPV) was 85.2% (95% CI: 77.1–91.3).

Given that 78.9% (60/76) of NDM-positive isolates were also positive for OXA-48-like, the association between OXA-48-like and CZA resistance was further evaluated after stratification by NDM status. Among NDM-negative isolates (*n* = 108), OXA-48-like was detected in 14/16 CZA-resistant and 85/92 CZA-susceptible isolates, and no significant association with CZA resistance was observed (OR = 0.576). A stratum-specific OR could not be estimated among NDM-positive isolates (*n* = 76), as all isolates were CZA-resistant. Consistently, Cochran–Mantel–Haenszel (CMH) analysis stratified by NDM status showed no significant association between OXA-48-like and CZA resistance (common OR = 0.576; CMH *p* = 0.515).

The performance of alternative gene combinations to NDM in predicting CZA resistance was evaluated. When NDM alone was used, the sensitivity was 82.6%, specificity was 100.0%, PPV was 100.0%, and NPV was 85.2%. When positivity for either NDM or KPC was considered panel positivity, sensitivity increased to 84.8%, whereas specificity decreased to 92.4% and PPV to 91.8%; NPV was 85.9%. When positivity for either NDM or OXA-48-like was considered panel positivity, sensitivity increased to 97.8%, whereas specificity was 7.6%, PPV was 51.4%, and NPV was 77.8% (Table 2).

### 2.4. Amikacin and Colistin Susceptibility According to CZA Susceptibility Status

All CZA-resistant isolates were resistant to carbapenems, cephalosporins, β-lactam/β-lactamase inhibitor combinations, and fluoroquinolones, whereas susceptibility to amikacin and colistin was retained in some isolates. Therefore, comparative analyses between CZA-susceptible and CZA-resistant isolates were performed for amikacin and colistin.

Among the CZA-resistant isolates, 36 (39.1%) were susceptible and 56 (60.9%) were resistant to amikacin, whereas among the CZA-susceptible isolates, 63 (68.5%) were susceptible and 29 (31.5%) were resistant to amikacin. Amikacin resistance was significantly higher among CZA-resistant isolates than among CZA-susceptible isolates (60.9% vs. 31.5%; *p* < 0.001), and this difference remained statistically significant after FDR correction (*q* < 0.001).

Colistin resistance was detected in 33/92 (35.9%) CZA-resistant isolates and 20/92 (21.7%) CZA-susceptible isolates. The difference between the groups yielded a *p* value of 0.050 in the unadjusted analysis. Following FDR correction, the result did not meet the prespecified significance threshold of *q* < 0.05 (*q* = 0.050). Among the 184 isolates with available colistin minimum inhibitory concentration (MIC) values, the median MIC was 2 µg/mL (interquartile range [IQR]: 1–4). The median colistin MIC was 2 µg/mL (IQR: 1–2) among CZA-susceptible isolates and 2 µg/mL (IQR: 1–8) among CZA-resistant isolates. No statistically significant difference in colistin MIC distributions was observed between CZA-susceptible and CZA-resistant isolates (*p* = 0.084).

## 3. Discussion

This study demonstrated a clear association between CZA resistance and carbapenemase genotypes among carbapenem-resistant *K. pneumoniae* isolates. NDM was detected at a high frequency among CZA-resistant isolates but was absent from all CZA-susceptible isolates. In addition, all CZA-susceptible isolates carried a single carbapenemase gene, whereas approximately two-thirds of the resistant isolates carried multiple carbapenemase genes. The NDM + OXA-48-like combination was the most common profile in the resistant group, whereas the OXA-48-like-only profile was more frequently associated with CZA susceptibility.

The observed association between NDM and CZA resistance is consistent with the limited activity of avibactam against MBLs [13,18]. Recent clinical and in vitro studies have also reported high rates of CZA resistance among MBL-producing CRKP isolates and have identified aztreonam-based combinations as potential treatment options for infections caused by these organisms [13,18,19]. Similarly, Zhang et al. reported the presence of MBLs in 53.1% of CZA-resistant CRKP isolates and demonstrated that MBL production may contribute substantially to CZA resistance [20]. Reports from different centers showing high rates of CZA resistance among isolates carrying NDM or NDM + OXA-48-like are also consistent with the present findings [21]. These data suggest that rapid molecular detection of NDM may help predict CZA activity and that carbapenemase typing may support antimicrobial treatment decisions [19,22]. The high discriminatory performance of NDM for CZA resistance observed in this study further supports this interpretation.

The co-occurrence of multiple carbapenemase genes was one of the notable epidemiological findings of this study. While all CZA-susceptible isolates carried a single carbapenemase gene, two or more carbapenemase genes were detected in 68.5% of the resistant isolates, with NDM + OXA-48-like being the predominant profile. This distribution is generally consistent with data reported from Türkiye and other geographical regions; co-production of OXA-48/KPC and KPC/NDM has been reported in Türkiye, and the co-occurrence of OXA-48 + NDM has also been documented [8,23,24]. Global genomic data have likewise shown that NDM + OXA-48-like is the most common combination among CRKP isolates carrying two carbapenemases, accounting for 79.9% of such isolates [24]. From a clinical perspective, these carbapenemase combinations may be associated with more limited antimicrobial treatment options; in particular, combinations involving MBLs are expected to substantially limit CZA activity. Furthermore, some studies have reported that isolates carrying multiple carbapenemases may be associated with adverse clinical outcomes [24,25].

Although OXA-48-like was detected at high frequencies in both groups, the OXA-48-like-only profile was more frequently associated with CZA susceptibility. This finding is consistent with the OXA-48-endemic epidemiological setting in Türkiye and the inhibitory activity of avibactam against OXA-48-like serine β-lactamases. Recent studies have reported high CZA susceptibility among isolates producing OXA-48-like alone, reaching 100% in some series [22,26,27]. Nevertheless, the presence of OXA-48-like alone does not guarantee CZA susceptibility; reduced CZA susceptibility has been reported in OXA-48-like-carrying isolates and may be associated with additional β-lactamase alterations, permeability defects, and other resistance mechanisms [16,28]. In this study, the association between OXA-48-like and CZA susceptibility was further evaluated by stratifying the analysis according to NDM status. Importantly, the OXA-48-like-only profile analysis and the NDM-stratified analysis addressed different comparisons. The former compared isolates carrying OXA-48-like as the sole detected carbapenemase with those harboring other carbapenemase profiles, whereas the latter assessed OXA-48-like positivity regardless of additional carbapenemase genes within NDM-defined strata. Although the OXA-48-like-only profile showed a significant inverse association with CZA resistance (OR = 0.015; *p* < 0.001), the NDM-stratified analysis did not demonstrate a statistically significant association between overall OXA-48-like positivity and CZA resistance (CMH OR = 0.576; *p* = 0.515). These findings are not contradictory, as the two analyses evaluated different exposure definitions and comparison groups. Therefore, the association observed for the OXA-48-like-only profile should not be interpreted as equivalent to that of overall OXA-48-like positivity after stratification by NDM status. These results underscore the importance of considering both specific carbapenemase gene profiles and NDM co-carriage when interpreting CZA susceptibility.

In this study, the frequency of KPC was similar between the CZA-susceptible and CZA-resistant groups, suggesting that the presence of KPC alone is insufficient to explain the CZA resistance phenotype. Although CZA activity has been reported to be retained in most KPC-producing isolates, mechanisms associated with CZA resistance include KPC variants involving the Ω-loop region, increased *bla*KPC copy number or expression, and porin alterations [14,29,30]. These mechanisms may potentially have contributed to the resistance phenotype observed among CZA-resistant KPC-positive isolates in this study. However, because targeted sequencing or whole-genome sequencing, gene expression analysis, and porin analysis were not performed, these explanations remain speculative and cannot be confirmed from the present data.

Amikacin resistance was significantly higher among CZA-resistant isolates than among CZA-susceptible isolates. Although colistin resistance was numerically higher in the CZA-resistant group, no significant difference was observed between the groups in terms of colistin MIC distributions. The difference in amikacin resistance is consistent with studies reporting that CRKP isolates carrying multiple carbapenemases frequently exhibit additional resistance to aminoglycosides and other antimicrobial agents. In particular, low amikacin susceptibility has been reported among NDM-positive isolates [19,24]. Similarly, high rates of amikacin resistance have been reported among NDM + OXA-48 co-producing isolates, and in some series, all isolates carrying NDM + OXA-48-like were resistant to amikacin [21]. In contrast, the findings regarding colistin should be interpreted with greater caution. Although colistin resistance was numerically higher in the CZA-resistant group in this study, the difference did not reach statistical significance, and no significant difference in colistin MIC distributions was observed between the groups. In contrast, colistin resistance rates ranging from 58.3% to 83.5% have been reported in the literature, particularly among NDM + OXA-48 co-producing isolates [31,32]. The differences between the resistance rates observed in this study and those reported in the literature may be related to variations in the carbapenemase profiles of the isolates investigated, local epidemiological characteristics, and the distribution of additional molecular mechanisms contributing to colistin resistance. These findings suggest that CZA resistance or a specific carbapenemase profile alone may not be sufficient to predict colistin resistance and that colistin susceptibility should be assessed separately using phenotypic methods.

Taken together, these findings suggest that carbapenemase genotypes may contribute not only to molecular epidemiological surveillance but also to the prediction of the CZA susceptibility phenotype. Particularly in regions where OXA-48-like carbapenemases are endemic, the co-occurrence of NDM may be considered a molecular feature strongly associated with CZA resistance [7,25]. Therefore, although rapid molecular carbapenemase typing cannot replace phenotypic antimicrobial susceptibility testing, it may support antimicrobial treatment selection when interpreted alongside phenotypic susceptibility results, particularly when empirical or early targeted use of CZA is being considered.

This study has several limitations. First, it was conducted at a single center using archived isolates, which may limit the generalizability of the findings. Because equal numbers of CZA-susceptible and CZA-resistant isolates were deliberately included, the PPV and NPV estimates reflect the selected study population and should not be directly extrapolated to routine clinical settings with different CZA resistance prevalence. Furthermore, although the presence of carbapenemase genes was investigated, carbapenemase variant analysis, gene expression and copy-number changes, porin alterations, efflux-related mechanisms, and other potential determinants of CZA resistance were not evaluated. Therefore, the molecular basis of CZA resistance in isolates lacking an MBL, particularly KPC- or OXA-48-like-positive resistant isolates, cannot be definitively established from the present data. Finally, clinical outcomes were not assessed; therefore, potential associations between different molecular profiles and patient outcomes could not be evaluated.

## 4. Materials and Methods

### 4.1. Study Design and Bacterial Isolates

This retrospective, laboratory-based study included 184 archived, non-duplicate carbapenem-resistant *Klebsiella pneumoniae* isolates recovered from various clinical specimens at the Medical Microbiology Laboratory of Ankara Etlik City Hospital between 1 July 2023 and 1 February 2024. The study set comprised 92 CZA-susceptible and 92 CZA-resistant isolates. Routine laboratory records during the study period included repeated isolates and multiple isolates from the same patients; therefore, the exact total number of unique, non-duplicate CRKP isolates corresponding to the study eligibility criteria could not be reliably reconstructed retrospectively. Only one isolate per patient was included in the study, and duplicate isolates were excluded. Following routine diagnostic procedures, the isolates were stored in stock medium at −80 °C. Molecular analyses for carbapenemase genes were performed in June 2025 after ethics committee approval (Ethics Committee of Ankara Etlik City Hospital, protocol code: AESH-BADEK-2024-157; date of approval: 14 February 2024).

### 4.2. Bacterial Identification and Antimicrobial Susceptibility Testing

Species-level identification of the isolates was performed using the MALDI Biotyper^®^ Sirius system (Bruker Daltonics, Bremen, Germany). Antimicrobial susceptibility testing was performed using different methods depending on the antimicrobial agent. Ceftazidime–avibactam (CZA) susceptibility was determined by the disk diffusion method based on inhibition-zone diameters, whereas colistin susceptibility was assessed using the broth microdilution (BMD) method to determine minimum inhibitory concentrations (MICs). Susceptibility testing for all remaining antimicrobial agents, including carbapenems and amikacin, was performed using the BD Phoenix™ M50 automated system (Becton Dickinson, Sparks, NV, USA).

The original antimicrobial susceptibility results obtained during routine laboratory testing were retrieved from the laboratory information system. Subsequently, the recorded inhibition-zone diameters and MIC values were retrospectively reinterpreted according to the European Committee on Antimicrobial Susceptibility Testing (EUCAST) version 16.0 (2026) breakpoints [33]. The susceptibility classifications obtained following this retrospective reassessment were used for all analyses presented in the study.

Quality assurance procedures were performed as part of the laboratory’s routine quality management system. Internal quality control was conducted using standard reference strains, including *Escherichia coli* ATCC 25922 and *Klebsiella pneumoniae* ATCC 700603, in accordance with established laboratory procedures. In addition, the laboratory participated in external quality assessment programs, and quality control activities were performed according to the schedules and requirements of the respective programs.

### 4.3. Molecular Detection of Carbapenemase Genes

Genomic deoxyribonucleic acid (DNA) was extracted from the isolates using the Bio-Speedy^®^ Rapid Nucleic Acid Extraction Kit (Bioeksen, Istanbul, Türkiye) on the Zybio EXM3000 automated nucleic acid extraction system. One to three colonies from fresh cultures grown on solid medium were suspended in 200 µL of Bio-Speedy^®^ STL-B buffer and vortexed at maximum speed for 10 s. Subsequently, 15 µL of proteinase K and 200 µL of the prepared bacterial suspension were added to the first well of the extraction cartridge. Automated extraction was performed according to the manufacturer’s instructions, and approximately 80–100 µL of the resulting nucleic acid extract was transferred to a nuclease-free microcentrifuge tube and used for molecular analyses.

Carbapenemase genes were investigated using a multiplex real-time polymerase chain reaction (PCR) assay with the Bio-Speedy^®^ Carbapenem Resistance qPCR Kit (Bioeksen, Istanbul, Türkiye; Cat. No. BS-AR-CR-25/BS-AR-CR-100). According to the manufacturer’s data, the kit has a sensitivity of 94.9%, a specificity of 95.1%, and a limit of detection (LoD) of 50 copies/mL. The assay targeted KPC, NDM, VIM, IMP, OXA-23/OXA-58, OXA-48, and OXA-51 genes. CRE Oligo Mix was used for the detection of KPC, NDM, VIM, and IMP, whereas OXA Oligo Mix was used for the detection of OXA-51, OXA-23/OXA-58, OXA-48, and the internal control.

Each reaction was prepared in a total volume of 10 µL, consisting of 5 µL of 2× qPCR Mix, 2.5 µL of the corresponding Oligo Mix, and 2.5 µL of template nucleic acid. Positive controls for both oligonucleotide mixtures (PC-CRE and PC-OXA) and a no-template control (NTC) were included in each run.

Amplification was performed using the Bio-Rad CFX96 Real-Time PCR System (Bio-Rad Laboratories, Hercules, CA, USA). The thermal cycling protocol was performed according to the manufacturer’s instructions. Enzyme activation was initially performed at 52 °C for 3 min, followed by an initial high-temperature step at 95 °C for 10 s. This was followed by 12 touchdown cycles consisting of denaturation at 95 °C for 1 s and annealing/extension for 15 s, with the annealing temperature decreasing by 1 °C per cycle from 67 °C to 56 °C. Subsequently, 30 amplification cycles were performed, consisting of denaturation at 95 °C for 1 s and annealing/extension at 55 °C for 15 s.

PCR results were analyzed by visual evaluation of the amplification curves using Sigmoida software (version 9.2.28; Bioeksen). The validity of each run was assessed using the positive and negative controls provided with the kit. Samples were considered positive only when a typical sigmoidal amplification curve above the threshold was observed together with a Cq value ≤30. Samples showing atypical, borderline, or non-sigmoidal amplification curves were considered indeterminate, and the analysis was repeated from the nucleic acid extraction step onward before final interpretation. Samples showing no amplification were considered negative.

### 4.4. Classification of Carbapenemase Gene Profiles

Isolates were classified as carrying either single or multiple carbapenemase genes according to the number of carbapenemase genes detected. Isolates in which only one carbapenemase gene was detected were classified as single-gene carriers, whereas those in which two or more carbapenemase genes were detected were classified as multiple-gene carriers.

In addition, carbapenemase gene profiles were defined based on the detected genes and their combinations. These profiles were compared according to CZA susceptibility status.

### 4.5. Statistical Analysis

Statistical analyses were performed using IBM SPSS Statistics version 27.0 (IBM Corp., Armonk, NY, USA). Categorical variables were expressed as numbers and percentages [*n* (%)] and compared using Pearson’s chi-square test or Fisher’s exact test, as appropriate. Colistin MIC values were presented as medians and interquartile ranges (IQRs; 25th–75th percentiles), and MIC distributions between CZA-susceptible and CZA-resistant isolates were compared using the Mann–Whitney U test.

Associations between carbapenemase gene profiles and CZA resistance were assessed by calculating odds ratios (ORs) with 95% confidence intervals (CIs). ORs were not reported when finite estimates could not be obtained because of zero cells. The association between OXA-48-like and CZA resistance was further evaluated after stratification by NDM status using the Cochran–Mantel–Haenszel (CMH) method.

The performance of NDM and combined gene panels in predicting CZA resistance was assessed using sensitivity, specificity, positive predictive value (PPV), and negative predictive value (NPV), with 95% CIs calculated using the exact Clopper–Pearson method. The Benjamini–Hochberg false discovery rate (FDR) correction was applied separately to multiple comparisons, and adjusted *p* values were reported as *q* values. Statistical significance was defined as a two-sided *p* < 0.05 for unadjusted analyses and *q* < 0.05 for FDR-adjusted analyses.

## 5. Conclusions

In conclusion, this study demonstrated a clear association between CZA resistance and carbapenemase gene profiles among CRKP isolates. The presence of NDM, particularly the NDM + OXA-48-like combination, was strongly associated with CZA resistance, whereas the OXA-48-like-only profile was more frequently associated with CZA susceptibility. These findings suggest that carbapenemase gene profiles may contribute to predicting the CZA susceptibility phenotype, particularly in regions where OXA-48-like carbapenemases are endemic. The use of rapid molecular carbapenemase typing in conjunction with phenotypic antimicrobial susceptibility testing may support molecular surveillance and the selection of appropriate antimicrobial therapy.

## Figures and Tables

**Figure 1:**
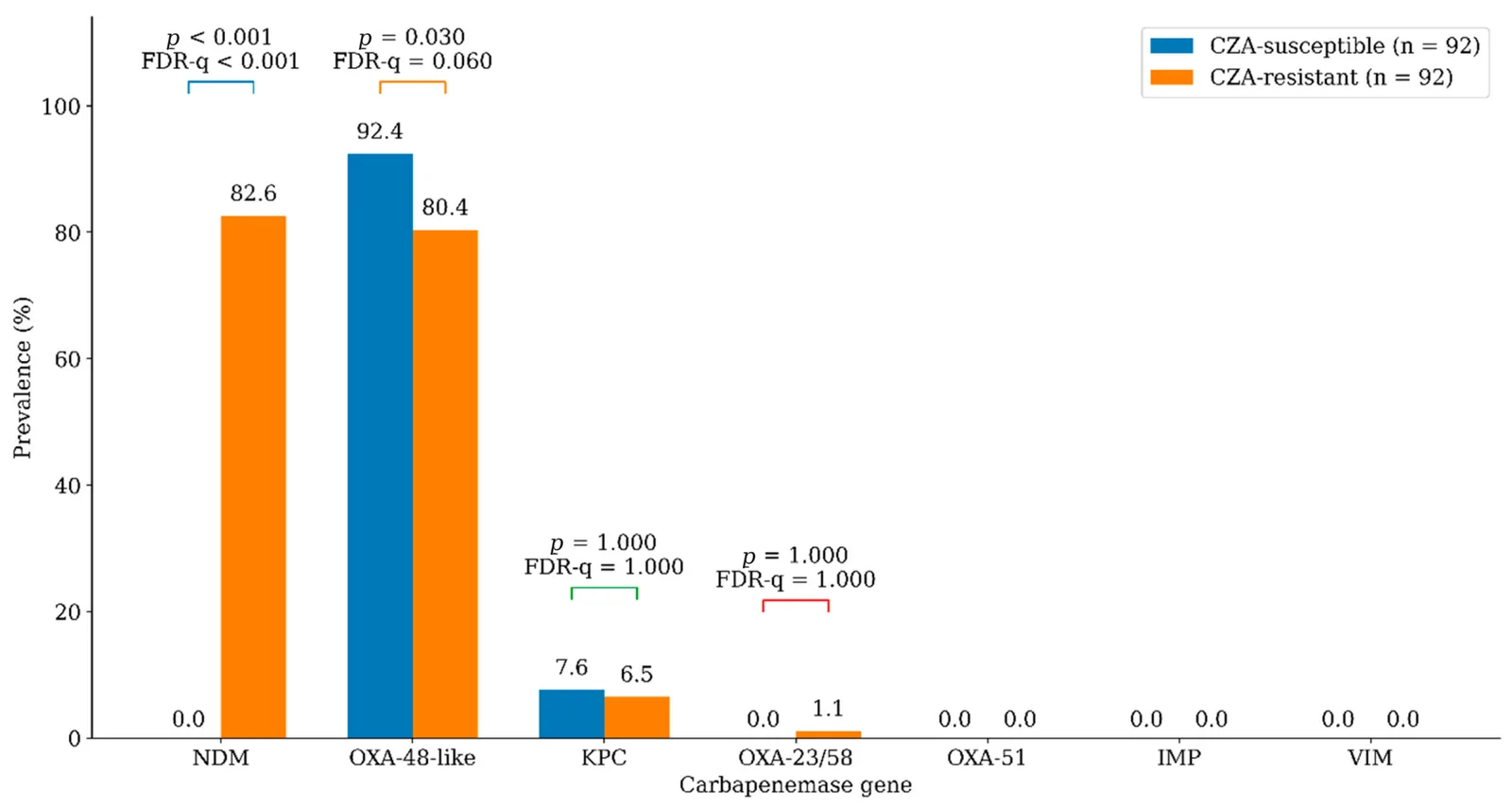
Distribution of carbapenemase genes according to ceftazidime–avibactam (CZA) susceptibility status. Comparisons between groups were performed using Fisher’s exact test. FDR-*q* values were calculated using the Benjamini–Hochberg procedure to adjust for multiple comparisons. FDR, false discovery rate.

**Table 1:** Association of Carbapenemase Gene Profiles with CZA Resistance.

Gene Profile	CZA-S *n* (%)	CZA-R *n* (%)	OR * (95% CI)	*p* Value ^†^	FDR-*q*
OXA-48-like only	85 (92.4)	14 (15.2)	0.015 (0.006–0.039)	<0.001	<0.001
KPC only	7 (7.6)	2 (2.2)	0.270 (0.055–1.335)	0.169	0.237
NDM only	0 (0.0)	13 (14.1)	NC	<0.001	<0.001
NDM + OXA-48-like	0 (0.0)	58 (63.0)	NC	<0.001	<0.001
NDM + KPC	0 (0.0)	3 (3.3)	NC	0.246	0.287
NDM + OXA-48-like + KPC	0 (0.0)	1 (1.1)	NC	1.000	1.000
NDM + OXA-48-like + OXA-23/58	0 (0.0)	1 (1.1)	NC	1.000	1.000

* The OR for the OXA-48-like-only profile was calculated by comparing isolates with this profile with isolates carrying other carbapenemase profiles. ^†^ Fisher’s exact test. FDR, false discovery rate. NC, not calculable due to zero cells in the 2 × 2 contingency table. CI, confidence interval; OR, odds ratio.

**Table 2:** Performance of Single and Combined Gene Panels in Predicting CZA Resistance.

Panel	Sensitivity, %	Specificity, %	PPV, %	NPV, %
NDM only	82.6	100	100	85.2
NDM or KPC	84.8	92.4	91.8	85.9
NDM or OXA-48-like	97.8	7.6	51.4	77.8

PPV, positive predictive value; NPV, negative predictive value. For combined gene panels, panel positivity was defined as positivity for at least one of the included genes. PPV and NPV were calculated based on the selected study population, which included equal numbers of CZA-susceptible and CZA-resistant isolates (50% resistance prevalence). Therefore, these estimates may not be directly generalizable to routine clinical populations with different resistance prevalence.

## Data Availability

Data supporting the findings of this study are available from the corresponding author: Elif Tuğçe Güner, upon reasonable request.

## Abbreviations

The following abbreviations are used in this manuscript:

CMH	Cochran–Mantel–Haenszel
CRKP	Carbapenem-resistant *Klebsiella pneumoniae*
CZA	Ceftazidime–avibactam
FDR	False discovery rate
IMP	Imipenemase
KPC	*Klebsiella pneumoniae* carbapenemase
MALDI-TOF MS	Matrix-assisted laser desorption/ionization time-of-flight mass spectrometry
MBL	Metallo-β-lactamase
MIC	Minimum inhibitory concentration
NDM	New Delhi metallo-β-lactamase
NPV	Negative predictive value
OXA	Oxacillinase
PPV	Positive predictive value
VIM	Verona integron-encoded metallo-β-lactamase
